# The association of arsenic exposure with mortality due to cancer, diabetes, Alzheimer's and congenital anomalies using Poisson regression

**DOI:** 10.1038/s41598-023-42744-4

**Published:** 2023-09-19

**Authors:** Alireza Rahmani, Samira Khamutian, Amin Doosti-Irani, Mohammad Javad Shokoohizadeh, Nasrin Shirmohammadi-Khorram, Fatemeh Sahraeei, Mahdi Khodabakhshi, Nastaran Ahangaran

**Affiliations:** 1 Department of Environmental Health Engineering, School of Public Health, Research Centre for Health Sciences, Hamadan University of Medical Sciences, Hamadan, Iran; 2 Department of Epidemiology, School of Public Health, Research Center for Health Sciences, Hamadan University of Medical Sciences, Hamadan, Iran; 3 Department of Environmental Health Engineering, School of Public Health, Kermanshah University of Medical Sciences, Kermanshah, Iran; 4 Department of Biostatistics, School of Public Health, Hamadan University of Medical Sciences, Hamadan, Iran; 5 Student Research Committee, Hamadan University of Medical Sciences, Hamadan, Iran; 6 Deputy of Public Health, Hamadan University of Medical Sciences, Hamadan, Iran

**Keywords:** Cancer, Environmental sciences, Environmental social sciences

## Abstract

The present study aims to determine the relationship between the concentration of arsenic in the groundwater of Hamadan province and the mortality rate due to various types of malignancies, congenital anomalies, diabetes mellitus and Alzheimer's. Mortality data due to various causes of death in Hamadan province were collected for five years (2016–2020). Sampling of drinking water was determined in the reference laboratory using polarography method. Poisson regression was used to investigate the relationship between arsenic level and the death rate due to various types of disease, at a significant level (p value < 0.05). According to the results of Poisson regression, among the various causes of death (N = 8042), Alzheimer's 5.94 (3.67–9.61), diabetes mellitus 4.05 (3.5–5.37), congenital malformations 2.98 (1.88–4.72), breast cancer 2.72 (1.56–4.71), leukemia 1.90 (1.24–2.92), stomach cancer 1.64 (1.28–2.10), Liver cancer 1.58 (1.58–2.30), other digestive organs 5.86 (3.38–10.16), meninges and brain cancer 1.57 (1.02–2.41) showed the highest relationship with arsenic contamination. The results of this study could be evidence for a positive and significant relationship between arsenic concentrations and mortality rates due to cancers, diabetes mellitus, Alzheimer disease, and congenital malformations. Therefore, it's necessary to use appropriate water treatment methods to remove arsenic at the source in contaminated areas.

## Introduction

Arsenic is one of the most important toxic elements that exist as organic and inorganic compounds in the environment^1^. Arsenic contamination is considered as a challenging threat to human health and sustainable agriculture. Pyrite minerals are one of the main natural sources of As in the environment, while anthropogenic sources include agricultural chemicals such as insecticides and herbicides, mining, manufacturing industries, coal burning, and chromated copper arsenate (CCA) wood preservatives^2^. Inorganic forms of arsenic, such as trivalent arsenite (As III) and pentavalent arsenate (As V) are the most widespread and toxic forms found in groundwater^3^. In the underground water of more than 70 countries, arsenic contamination has been reported, and affected the health of millions of people. The countries which are at the top of list for As contamination in groundwater are: China, Chile, India, Cambodia, Argentina, America, Taiwan, Bangladesh, Austria, Vietnam, Hungary, Japan, Nepal, Canada, and Iran^4^. Arsenic is absorbed by the body through water, air, contaminated food, skin, respiratory system and digestive system, and then it is widely revealed in the blood stream and spreads in a large volume of body tissues and causes various diseases. The increasing acute and chronic effects of arsenic on human health have led to the classification of arsenic as a class I human carcinogen by the International Agency for Research on Cancer (IARC)^5^. The continuous use of groundwater contaminated with this element for drinking and irrigation has led to numerous health manifestations in the human body, which is generally called arsenicosis. These complications include intestinal, kidney, lung and bladder disorders, skin effects and cancer in different organs. Arsenic toxicity also causes neurological dysfunction, cardiovascular disease, and immune-related disorders^6^. Arsenic consumption through water and food can cause reproductive problems in women and men, and it's also associated with childbirth issues such as stillbirth, abortion and premature birth. Reports show that children in areas with high arsenic contamination, have a higher risk of malnutrition, severe weight loss^7^. Based on the results of the analysis of water samples of Hamadan province, the groundwater of some areas, especially in Kabudarahang, is contaminated with arsenic. Considering the known effects of exposure to arsenic through drinking water, the present study aims to determine the relationship between the As concentration in the groundwater and the death rate due to malignancies, diabetes mellitus and congenital anomalies, Alzheimer's in the contaminated areas compared to other regions.

## Results

**Figure 1:**
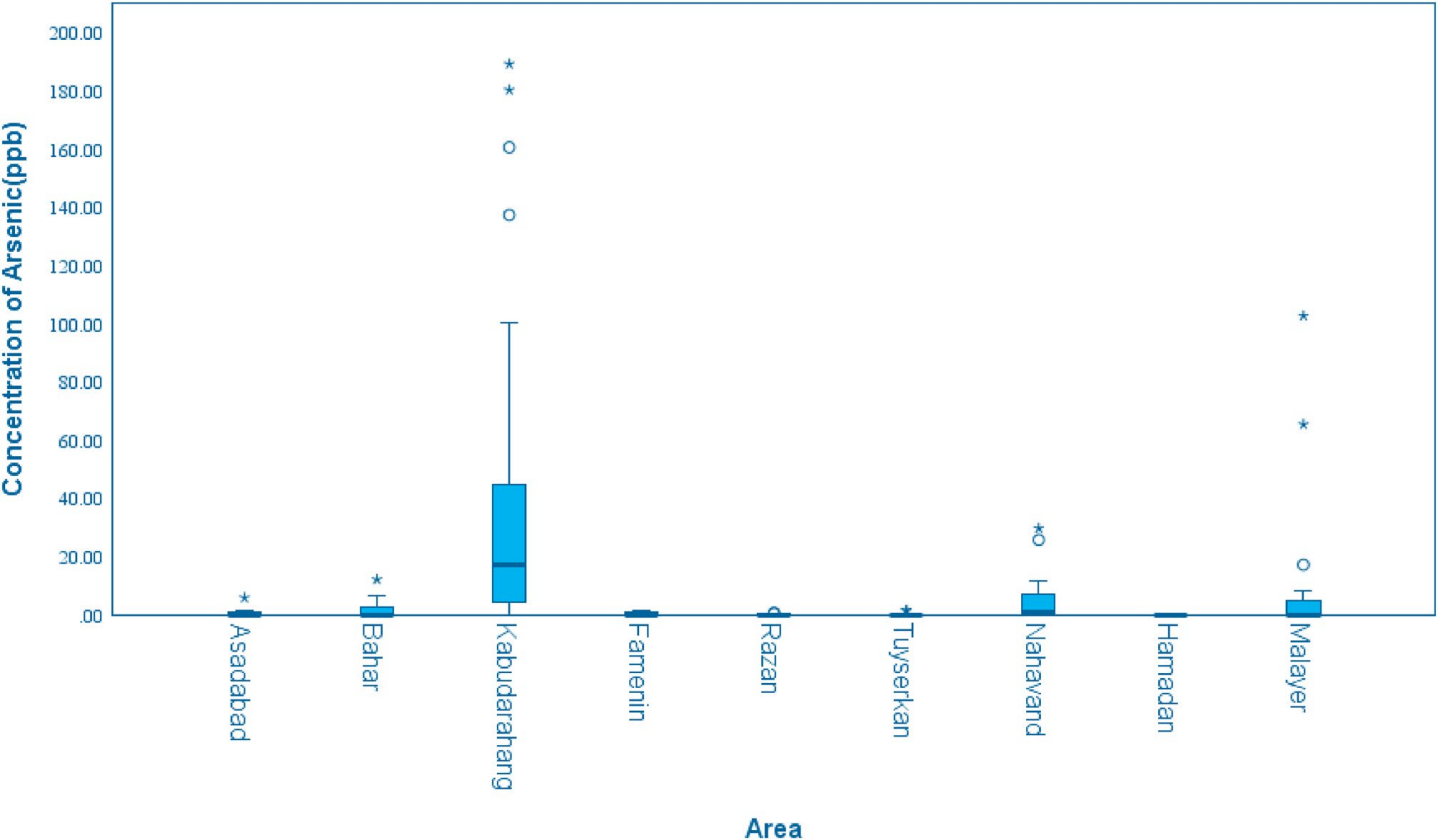
Box plots of arsenic concentration in the nine areas of Hamadan province.

The highest contamination of groundwater is reported in the rural areas of Kabudarahang with a maximum concentration of 190 µg per liter. In some areas of Malayer, Nahavand and Kabudahang, the concentration of arsenic was 2 to 18 times higher than the maximum residue limit (MRL). Table 1 and Fig. 1 give summary statistics and box plot for arsenic concentration in the nine areas, respectively.

**Table 1:** Summary statistics of concentration of arsenic for each county.

Cities	Mean	Min	Max	Std. deviation	Kurtosis		Skewness	
					Statistic	Std. error	Statistic	Std. error
Asadabad	0.36	.00	1.60	.571	1.65	0.71	1.97	1.40
Bahar	2.05	.00	12.25	3.64	1.94	0.58	3.47	1.12
Kabudarahang	40.1	.00	180	20.72	1.83	0.37	2.52	0.74
Famenin	0.36	.00	1.41	0.59	1.37	0.75	.059	1.48
Razan	0.14	.00	.65	.26	1.47	0.637	.39	1.23
Tuyserkan	0.28	.00	1.67	.600	1.98	0.63	2.51	1.23
Nahavand	6.1	.00	29.81	10.32	1.77	0.616	1.97	1.19
Hamadan	0.00	.00	0.00	.000				
Tuyserkan	0.28	.00	1.67	.600	1.98	0.63	2.51	1.23
Malayer	13.10	.00	102.80	30.057	2.55	0.58	5.99	1.12

Table 2 represents the death rate (per 100,000 people) due to various types of cancer, congenital anomalies, diabetes and Alzheimer's for each county during 2016 to 2020. According to the findings in the villages of Kabudarahang, the highest death rate during 2016–2020 related to diabetes mellitus, stomach cancer, bronchus and lung cancer, Alzheimer's, leukemia, brain and meninges cancer, and congenital anomalies, respectively.

**Table 2:** The death rate (per 100,000 people) due to various types of disease for each county during 2016 to 2020.

name of disease	kabudarahang	Bahar	Malayer	Nahavand	Tuyserkan	Asadabad	Famenin	Razan	Hamadan
**Rural regions**									
Oesophagus	8.91	4.98	9.96	15.23	13.20	0.00	11.93	12.42	12.17
Stomach	75.23	38.17	81.64	66.80	118.80	4.74	43.74	103.05	58.80
Small intestine	3.96	14.94	11.95	2.34	6.60	0.00	7.95	6.21	0.00
Colon	17.82	8.30	28.87	18.75	55.00	0.00	15.90	50.91	29.40
Liver	31.67	39.83	26.88	16.41	50.60	0.00	15.90	45.94	33.45
Gallbladder	0.99	3.32	2.99	0.00	4.40	0.00	0.00	1.24	0.00
Pancreas	1.98	6.64	7.96	10.55	8.80	0.00	11.93	12.42	10.14
Other digestive organs	21.78	8.30	8.96	0.00	11.00	0.00	15.90	3.72	4.06
Larynx	8.91	3.32	13.94	21.09	19.80	0.00	19.88	17.38	12.17
Bronchus and lung	35.63	14.94	46.79	41.02	55.00	7.12	15.90	58.36	53.73
Bone	0.99	6.64	0.00	3.52	2.20	0.00	3.98	4.97	10.14
Skin	4.95	4.98	4.98	0.00	0.00	0.00	0.00	3.72	11.15
Skin of scalp and neck	0.99	0.00	0.00	0.00	0.00	0.00	0.00	0.00	2.03
Breast	16.83	1.66	11.95	11.72	2.20	0.00	3.98	9.93	17.23
Cervix uteri, uteri	0.99	1.66	5.97	4.69	2.20	0.00	0.00	8.69	5.07
Ovary	1.98	1.66	4.98	1.17	0.00	0.00	0.00	2.48	4.06
Prostate	7.92	1.66	31.86	32.81	48.40	2.37	19.88	32.28	23.32
Kidney, renal pelvis	1.98	3.32	2.99	1.17	13.20	2.37	0.00	3.72	4.06
Bladder	6.93	6.64	14.93	5.86	11.00	4.74	3.98	8.69	4.06
Meninges, brain	24.75	24.90	20.91	14.06	35.20	4.74	11.93	13.66	47.65
Spinal cord	0.00	1.66	0.00	0.00	0.00	0.00	0.00	0.00	4.06
Lymphoma	1.98	3.32	0.00	2.34	4.40	0.00	0.00	4.97	5.07
Myeloma	2.97	3.32	2.99	2.34	8.80	0.00	0.00	0.00	2.03
Leukaemia	25.74	21.58	26.88	4.69	30.80	4.74	15.90	27.32	23.32
Other lymphoid	8.91	0.00	3.98	24.61	8.80	0.00	19.88	16.14	6.08
Diabetes mellitus1	1.98	19.92	3.98	12.89	4.40	0.00	23.86	37.25	5.07
Diabetes mellitus(2)	8.91	8.30	30.86	21.09	4.40	0.00	31.81	0.00	7.10
Unspecified diabetes mellitus	72.26	21.58	1.00	3.52	57.20	0.00	11.93	24.83	79.07
Diabetes mellitus	84.13	48.13	39.82	37.50	68.20	0.00	67.59	67.05	95.29
Congenital malformations	24.75	11.62	18.92	14.06	33.00	2.37	3.98	8.69	5.07
Alzheimer disease	28.70	4.98	1.99	1.17	0.00	0.00	3.98	29.80	8.11
**Urban regions**									
Oesophagus	3.99	3.40	6.91	4.35	3.57	1.71	0.00	22.18	11.22
Stomach	59.92	17.00	31.36	59.80	28.54	6.84	70.38	25.88	43.12
Small intestine	3.99	1.70	1.59	2.17	3.57	0.00	0.00	0.00	2.10
Colon	31.96	6.80	15.94	19.57	19.62	0.00	35.19	11.09	32.61
Liver	7.99	13.60	27.64	16.31	26.76	0.00	0.00	11.09	23.31
Gallbladder	3.99	1.70	1.06	3.26	1.78	0.00	14.08	0.00	3.68
Pancreas	3.99	15.30	12.76	13.05	14.27	3.42	7.04	3.70	19.63
Other digestive organs	7.99	5.10	14.88	0.00	7.13	0.00	0.00	3.70	2.63
Larynx	0.00	8.50	2.66	7.61	5.35	0.00	14.08	11.09	7.19
Bronchus and lung	39.95	40.79	36.14	46.75	32.11	5.13	7.04	29.58	37.86
Bone	7.99	3.40	1.59	1.09	1.78	0.00	0.00	0.00	5.08
Skin	0.00	5.10	2.13	1.09	0.00	3.42	0.00	0.00	4.21
Skin of scalp and neck	0.00	0.00	0.53	0.00	0.00	0.00	0.00	0.00	0.53
Breast	27.96	13.60	19.66	17.40	17.84	3.42	7.04	11.09	25.42
Cervix uteri, uteri	3.99	0.00	6.91	3.26	10.70	0.00	0.00	7.39	5.08
Ovary	3.99	1.70	5.31	3.26	1.78	3.42	0.00	0.00	11.57
Prostate	7.99	6.80	18.60	16.31	7.13	3.42	0.00	3.70	23.14
Kidney, renal pelvis	0.00	1.70	2.13	1.09	7.13	0.00	7.04	0.00	4.91
Bladder	7.99	10.20	11.16	13.05	8.92	8.56	0.00	3.70	11.92
Meninges, brain	0.00	13.60	17.01	9.78	35.67	0.00	14.08	11.09	27.35
Spinal cord	0.00	0.00	0.00	0.00	0.00	0.00	0.00	0.00	0.88
Lymphoma	7.99	0.00	2.13	3.26	7.13	1.71	0.00	3.70	6.14
Myeloma	0.00	8.50	6.38	2.17	0.00	0.00	7.04	11.09	7.19
Leukaemia	19.97	18.70	13.29	6.52	26.76	5.13	0.00	18.49	20.86
Other lymphoid	0.00	1.70	13.29	18.48	14.27	0.00	21.11	11.09	3.51
Diabetes mellitus1	3.99	11.90	13.82	7.61	3.57	0.00	14.08	18.49	8.24
Diabetes mellitus(2)	0.00	6.80	14.35	20.66	1.78	3.42	42.23	3.70	11.04
Unspecified diabetes mellitus	79.89	11.90	3.72	4.35	32.11	0.00	14.08	36.97	75.55
Diabetes mellitus	83.89	32.30	32.42	32.62	39.24	3.42	70.38	55.46	94.49
Congenital malformations	27.96	11.90	15.94	13.05	41.03	1.71	0.00	14.79	16.65
Alzheimer disease	7.99	5.10	0.53	0.00	16.05	0.00	0.00	11.09	20.51

According to the results of Poisson regression (Table 3), among the various causes of death (N = 9473), Alzheimer disease 5.94 (3.67–9.61), diabetes mellitus 4.05 (3.5–5.37), congenital malformations 2.98 (1.88–4.72), and cancer of breast cancer 2.72 (1.56–4.71), leukemia 1.90 (1.24–2.92), stomach cancer 1.64 (1.28–2.10), Liver cancer 1.58 (1.58–2.30), other digestive organs 5.86 (3.38–10.16), meninges, and brain cancer 1.57 (1.02–2.41) showed the highest relationship with arsenic pollution at a significant level (p-value < 0.05).

**Table 3:** Evaluation of relation between concentration of arsenic and death rate due to various disease using Poisson regression.

No	Type disease	IRR*	P_value	[95% conf. interval]
1	Stomach, C16.9, C16.8, C16	1.64	< 0.001	1.28–2.10
2	Other lymphoid, C96–C96.9	1.35	0.395	0.67–2.75
3	Oesophagus C15, C15.9	1.26	0.51	0.62–2.55
4	Skin of scalp and neck, C44.4	4	0.25	0.36–44
5	Bone, C41, C41.2, C41.8, C41.9	0.34	0.30	0.04–2.57
6	Breast, C50, C50.8, C50.9	2.72	< 0.001	1.56–4.71
7	bronchus and lung, C34, C34.1, C34.8, C34.9	1.29	0.15	0.90–1.83
8	cervix uteri, uteri, C53, C53.9, C54, C54.1, C54.9, C55	0.33	0.28	0.04–2.46
9	Colon, C18, C18.8, C18.9, C19, C20, C21	0.96	0.89	0.59–1.57
10	Gallbladder, C23	1	1	0.12–7.99
11	Kidney, renal pelvis, C64, C65	0.8	0.76	0.18–3.42
12	Larynx, C32, C32.9	0.97	0.93	0.48–1.94
13	Myeloma, C90, C90.0	1.84	0.33	0.52–6.47
14	other digestive organs, C26, C26.0, C26.9	5.86	< 0.001	3.38–10.16
15	Ovary, C56	1.23	0.78	0.27–5.45
16	Pancreas, C25, C25.0, C25.9	0.33	0.12	0.08–1.37
17	Prostate, C61	0.46	0.03	0.22–0.94
18	Skin, C43.9, C44, C44.9, C44.7	1.81	0.22	0.68–4.80
19	small intestine, C17, C17.1, C17.9	0.96	0.95	0.34–2.73
20	spinal cord, C72.0, C72.1	2.07e − 07	0.99	0.00
21	Bladder, C67, C67.9	1.30	0.51	0.58–2.89
22	Leukaemia, C90.1–C95.9	1.90	0.00	1.24–2.92
23	Liver, C22, C22.0, C22.1, C22.4, C22.7, C22.9	1.58	0.01	1.08–2.30
24	Lymphoma, C81, C81.9, C83, C83.0, C83.9, C85.9, C84.4, C85.1	1.06	0.93	0.24–4.66
25	meninges, brain, C70, C70.9, C71, C71.0, C71.9	1.57	0.03	1.02–2.41
26	Alzheimer disease, G30–G30.9	5.94	< 0.001	3.67–9.61
27	Congenital malformations, Q03.9–Q90.9	2.98	< 0.001	1.88–4.72
28	Unspecified diabetes mellitus(diabetes mellitus1 + (diabetes mellitus2), E10, E10.2, E10.7, E10.9, E11–E11.9, E14–E14.9	4.05	< 0.001	3.06–5.37

*Incidence rate ratio, computed for Kabudarahang in comparison of the other eight cities.

According to the results of Table 4, the risk of death due to Alzheimer disease (68%), diabetes mellitus (68%), Congenital malformations (58%) and cancers of ovary (83%), myeloma (78%), gallbladder (71%), breast (76%), pancreas (69%), lymphoma (67%), bladder (60%), cervix uteri (50%) kidney (43%), meninges and brain cancer (39%), other digestive organs (37%), colon (37%), leukemia (36%), bronchus and lung (36%), oesophagus (32%), Prostate (21%) and Liver (21%) in urban regions was higher than that in rural regions at a significant level (p value < 0.05). However, the risk of death due to the small intestine in urban regions was 76% lower than rural area.

**Table 4:** Comparison of death rate due to various disease in rural and urban using Poisson regression.

No	Type disease	IRR*	P_value	[95% Conf. interval]
1	Stomach, C16.9, C16.8, C16	0.98	0.84	0.85–1.13
2	Other lymphoid, C96–C96.9	0.774	0.152	0.54–1.09
3	Oesophagus C15, C15.9	0.68	0.02	0.49–0.95
4	Skin of scalp and neck, C44.4	0.56	0.5	0.1–3.07
5	Bone, C41, C41.2, C41.8, C41.9	0.68	0.14	0.40–1.14
6	Breast, C50, C50.8, C50.9	0.24	< 0.001	0.18–0.33
7	bronchus and lung, C34, C34.1, C34.8, C34.9	0.64	< 0.001	0.54–0.75
8	cervix uteri, uteri, C53, C53.9, C54, C54.1, C54.9, C55	0.5	0.005	0.30–0.80
9	Colon, C18, C18.8, C18.9, C19, C20, C21	0.63	< 0.001	0.51–0.77
10	Gallbladder, C23	0.29	0.002	0.13–0.63
11	kidney, renal pelvis, C64, C65	0.57	0.04	0.33–0.98
12	Larynx, C32, C32.9	1.26	0.17	0.90–1.75
13	Myeloma, C90, C90.0	0.22	< 0.001	0.12–0.41
14	other digestive organs, C26, C26.0, C26.9	0.63	0.04	0.40–0.99
15	Ovary, C56	0.17	< 0.001	0.09–0.31
16	Pancreas, C25, C25.0, C25.9	0.31	< 0.001	0.23–0.43
17	Prostate, C61	0.79	0.04	0.64–0.99
18	Skin, C43.9, C44, C44.9, C44.7	0.72	0.24	0.42––1.24
19	small intestine, C17, C17.1, C17.9	1.76	0.04	1.02
20	spinal cord, C72.0, C72.1	1.12	0.85	0.32–3.88
21	Bladder, C67, C67.9	0.40	< 0.001	0.28–0.57
22	Leukaemia, C90.1–C95.9	0.64	< 0.001	0.51–0.82
23	Liver, C22, C22.0, C22.1, C22.4, C22.7, C22.9	0.79	0.02	0.65–0.97
24	Lymphoma, C81, C81.9, C83, C83.0, C83.9, C85.9, C84.4, C85.1	0.33	< 0.001	0.18–0.60
25	meninges, brain, C70, C70.9, C71, C71.0, C71.9	0.61	< 0.001	0.50–0.77
26	Alzheimer disease, G30–G30.9	0.32	< 0.001	0.22–0.46
27	Congenital malformations, Q03.9–Q90.9	0.42	< 0.001	0.31–0.55
28	Unspecified diabetes mellitus(diabetes mellitus1 + (diabetes mellitus2), E10, E10.2, E10.7, E10.9, E11–E11.9, E14–E14.9	0.32	< 0.001	0.26–0.39

*Incidence rate ratio, computed for rural in comparison of urban.

## Discussion

### The results of arsenic concentration in the groundwater of the studied area

Based on the results, the highest level of arsenic contamination has been observed in the rural areas of Kabudarahang, which is located in the northwest of Hamadan province and adjacent to Kurdistan and Zanjan provinces. According to the studies, the origin of arsenic contamination in these areas is geological and geogenic activities. Chemical weathering of high altitudes such as volcanic and metamorphic rocks usually leads to the production of iron oxide. This weathering also causes the release of arsenic, which is absorbed on iron oxide or precipitates. An increase in pH in the area can cause the release of arsenic from iron oxide^8^. Arsenic competes with other ligands in groundwater for absorption on the surface of aquifer materials. Ligands that are usually found in high concentrations in groundwater (such as silicates, carbonates and organic acids) significantly reduce arsenic absorption^9^. In the studies conducted in the past on the groundwater of some regions of Iran, including the northwest of Iran (West Azerbaijan, East Azerbaijan, Kurdistan), the concentration of arsenic has been reported to exceed the standard limit^10,11^. In a study by Keshavarzi et al.^11^ in Kurdistan province, the range of arsenic concentration in underground water was 119–310, and the average concentration of arsenic was 193 µg/L^11^. In another study by Mosaferi et al.^12^ in Ardabil province, the range of arsenic concentration in urban water samples was 6–61 µg/L and the average concentration of arsenic was 39 µg/L^12^. In a study by Sadeghi in Ardabil, the arsenic concentration exceeded the standard for 70% of water samples collected during four different seasons^13^. In East Azerbaijan, the range of arsenic concentration of underground water was found in the range of 3 to 150 µg/L with an average of 80 µg/L^10^. About 230 million people from 108 countries are affected by arsenic contamination in groundwater, and 180 million of this population belongs to Asian countries^4^. The top list of countries with elevated arsenic content of groundwater includes India^14^, Bangladesh^15^, Nepal^16^, China^17^, and Vietnam^18^. In addition, countries such as South Africa, Argentina, Chile, Hungary, Canada, Pakistan, and Mexico and Iran have also been affected by arsenic contamination^19^.

### The relationship between mortality and arsenic concentration in groundwater

The findings of the present study showed that there is a positive and significant relationship between arsenic concentration and mortality due to cancers of the breast, meningeal and brain, stomach, liver, other digestive organ, and leukemia. In previous studies, there has been an association between arsenic in drinking water and mortality due to various cancers, which is consistent finding with the results of the present study. For example, in a meta-analysis study by Wang et al., a significant association between long-term exposure to arsenic and liver cancer mortality was observed (SMR, 1.84; 1.45–2.24). The final results of that study were obtained based on the analysis of 12 ecological and cohort articles conducted in Asia and South America^20^. A systematic review study by Khanjani et al., was conducted on the relationship between breast cancer and arsenic, in which seven studies were selected at the end, and a positive and significant association between arsenic and breast cancer was observed in four studies, while in the other three studies such a relationship was not seen. The reason for the difference in the results can be due to individual and regional differences^21^. The results of a study by Chen et al., in Taiwan showed a significant positive correlation between leukemia and arsenic in village well waters and arsenic concentration )SMR, 1.42; 1–1.84)^22^. A cross-sectional study in Bengal showed that long-term exposure to arsenic in people drinking contaminated water increased lymphocyte levels by 3.5 times compared to the control group of individuals^23^. On the other hand, in a study by Lin et al., a negative relationship was observed between the incidence of leukemia and arsenic levels in men and women^24^. In another study conducted in Chile, although a significant relationship was observed mortality due to liver cancer and arsenic, no significant relationship between was observed, no significant relationship was observed with arsenic level regarding mortality due to leukemia and brain cancer^25^. Compared to other cancers, fewer studies have investigated the relationship between arsenic and cancers of the stomach and other digestive organs have been investigated compared to other cancers. In a study conducted in southern Taiwan, the SMR for cancers of the stomach, colon, liver, bone, skin, and bladder was significantly higher in men and women who lived in the contaminated area than those in the non-contaminated area^26^.

In the present study, a significant relationship was found between arsenic exposure and mortality due to Alzheimer's disease. Convincing evidence suggests that arsenic causes such as neurotoxicity and impairment of memory and cognition. In addition, arsenic can cause pathological processes including mitochondrial disorders, oxidative stress, inflammation, apoptosis, abnormal calcium signaling, and disruption of protein homeostasis. However, the hypothesis and molecular mechanisms of arsenic-related pathology in Alzheimer are still unclear^5^. In an ecological study by Li et al.^27^, the association between exposures to contaminated soil and the death rate due to Alzheimer was investigated, and the relative risk (RR) and Spearman's correlation coefficient were calculated using based on10-year Alzheimer's mortality data. Based on the results, in the areas where the average concentrations of arsenic were 9.05, 10.4, and 13.1 μg/L, relative risks were calculated and found to be 4.3 (CI 4.3–4.39), 6.1 (CI 6.04–6.17), and 9.1 (CI 9.03–9.2), respectively^27^.

In the present study, a significant relationship between arsenic and there was a significant relationship between arsenic exposure and mortality due to congenital anomalies. Previous reports have shown that long term exposure to arsenic through drinking water may cause reproductive problems in women and men and is associated with various issues related to childbirth, such as stillbirth, miscarriage, premature birth, and the congenital abnormalities^7^. In a study by Marie et al. in France, 5263 children who were exposed to high concentration of arsenic in drinking water were assessed, and a positive and significant association was found between exposures to As concentration greater than 10 μg/L and the risk of congenital malformation (OR 2.41; CI 1.36–4.14)^28^. In another study, Ahmad et al. investigated the relationship between maternal exposure to arsenic through drinking water and the rate of miscarriage and as well as infant death. Based on the results, the risks of mortality as a combination of abortion and infant death for each unit of logarithmic increase of arsenic concentration equal corresponded to 1.35 (CI1.69, 1.08) for weeks 25 to 28 and 0.81 (CI1.02, 0.65) respectively, for weeks 9–12^29^.

In the present study, deaths due to diabetes mellitus showed a significant relationship with high levels of arsenic in contaminated areas. Previous animal studies and human evidences support a potential role of early-life exposure to arsenic in the development of diabetes, possibly by increasing insulin resistance^30^. In an ecological study by Mahram et al., in Qazvin province, the prevalence of type 1 diabetes and hypertension were investigated in two cities with different arsenic concentrations, in the range of 20–30 µg/L and less than 5 µg/L. Based on the results, there was a significant correlation between arsenic concentration and prevalence of diabetes and hypertension (p < 0.001), and the average prevalence of hypertension in relatively high contaminated and areas with lower contamination was obtained as 7.09% and 3.73%, and for diabetes was 53.4% and 1.99%, respectively^31^. In a community-based study in Canada (where diabetes rates continue to climb), the incidence rate of type 1 diabetes in the age group (0–14) was 51.7 per 100,000 people, and a significant relationship was observed between the incidence of type 1 diabetes and concentration of arsenic and fluoride with the having correlation coefficient (β = 0.268, p < 0.013,) and (β = 0.202, p < 0.005) respectively^32^. In another study by Marie et al., in France, the relationship between exposure to arsenic in drinking water and the risk of Gestational Diabetes was investigated in an ecological study, while the prevalence of gestational diabetes during the years 2003, 2006 and 2010 was analyzed using multivariate logistic regression. According to the findings, 5.7% of 5053 participants had gestational diabetes, and adjusted odds of developing gestational diabetes for women lived in the contaminated area (> 10 μg/L), were significantly higher than those who lived in the area with less than 10 μg/L (OR:1.62, CI 1.01–2.53)^33^.

According to the results of Poisson Regression, the death rate due to most of the cancers (including oesophagus, breast, bronchus and lung, uteri, colon, gallbladder, kidney, myeloma, ovary, pancreas, prostate, small intestine, other digestive organs, bladder, leukemia, liver, lymphoma, brain), Alzheimer’s Disease, diabetes mellitus 2, and congenital anomalies in urban regions were significantly higher than rural regions (p < 0.05).

Industrialization causes the transfer of the most important cause of death from infectious diseases and nutritional deficiency to chronic diseases (e.g., cancer and diabetes). According to the evidence, urbanization not only shows a direct effect on people's lifestyles and behavior but also affects the level of physical activity of people. Sedentary lifestyles and being inactive increase the risk of cancers, such as colon, breast, and prostate^34^. In addition, the highest smoking prevalence is among the socioeconomic groups and in urban areas that makes the higher lung cancer incidence in an urban population. Furthermore, air pollution is another factor in the development of lung cancer in urban areas^35^. On the other hand, based on the previous studies, there was a significant relationship between smoking, diabetes type2, obesity, over-weight and, physical inactivity, depression, hypercholesterolemia, hypertension and Alzheimer’s disease^36^.

The current findings complement a study by Zahnd et al.^37^ that showed combined cancers incidence rates were generally higher in urban populations. For individual cancers, urban regions had higher rates of prostate, breast (female), and thyroid cancers. However, cancer rates associated with modifiable risks-HPV, tobacco, and some preventive screening modalities (e.g., cervical cancers and colorectal) were higher in rural compared with urban populations^37^.

This study has two limitations; First, as mentioned earlier, there are several types of arsenic, including As5 + , As3 + , MMA, and DMA that adversely affects human health, however, in the present study, the concentration of different types of arsenic has not been determined separately. Second, some disruptive factors such as obesity, diet, physical activity, smoking, and genetic factors, which can contribute to disease risk, haven’t been assessed in this study. Identifying cancer risk factors plays an important role in preventing and controlling them in each region. In this regard, it is suggested that further studies be designed to consider all confounding factors and assess health effects of the different type of arsenic species.

The results of this study could be evidence for a strong positive association of arsenic in drinking water and mortality rate due to cancers of the breast, liver, stomach, meninges and brain, congenital anomalies, diabetes and Alzheimer's disease. Considering various health complications resulting from the consumption of arsenic-contaminated water, it's vital to determine the amount of arsenic in water sources before implementing any water supply program, and use appropriate water purification methods to remove arsenic of water resources, or use of alternative water sources.

## Materials and methods

**Figure 2:**
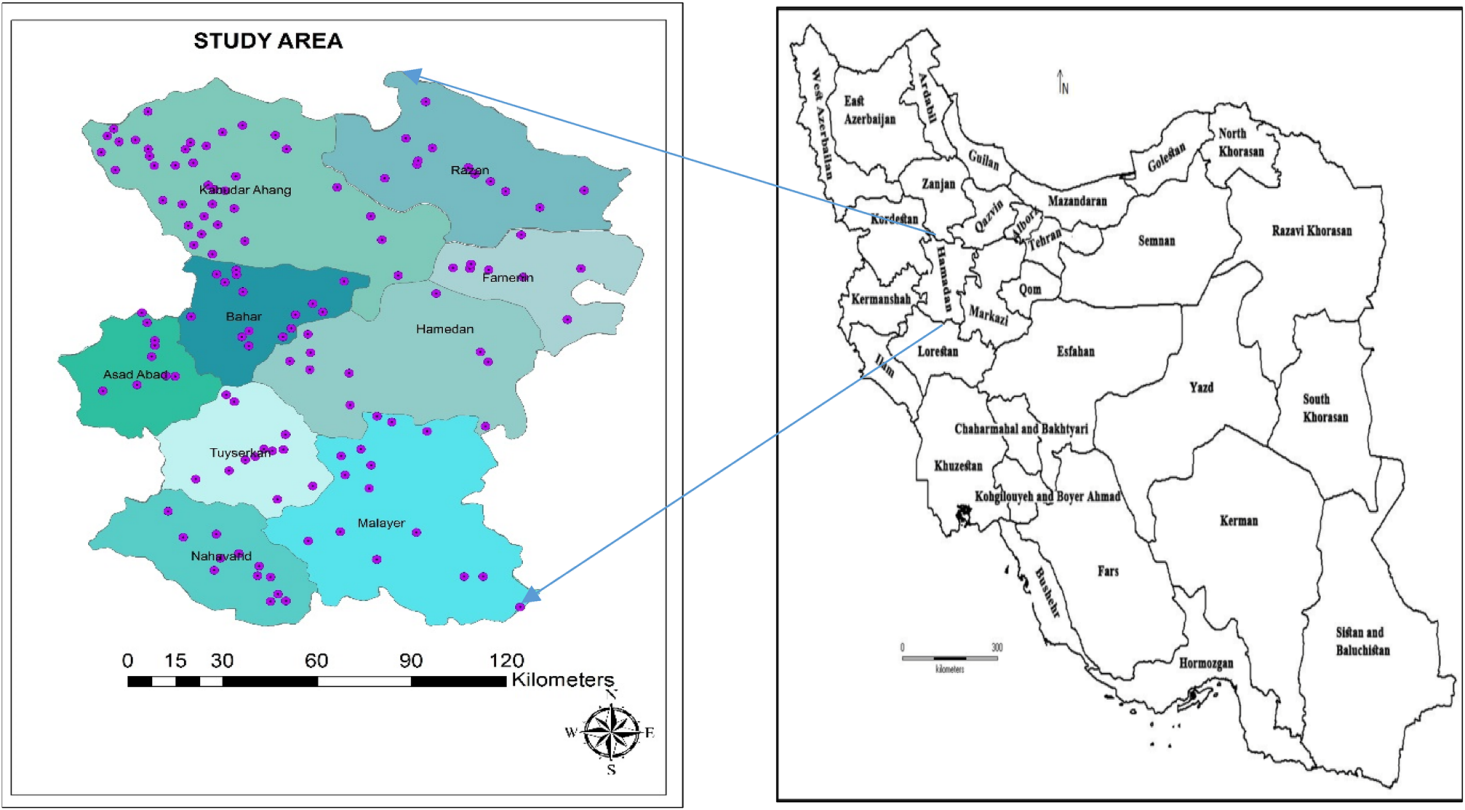
Maps of the study area and sampling location.

The places in the study include; Asadabad, Bahar, Famenin, Hamadan, Malayer, Nahavand, Razan, Tuyserkan, and Kabudarahang, which are nine parts of Hamadan province. Hamadan Province, situated between latitudes 33º59′ N and 35º48′ N and longitudes 47º34′ E and 49º36′ E, covering an area of approximately 19,546 km^2^ (as shown in Fig. 2), and its population is around 1,738,234 population^38^. The climate of Hamadan is typically arid or semi-arid and characterized by long, cold winters and mild summers. The average temperatures in the hottest month (July) and the coldest month (January) are 25 °C and − 2 °C, respectively. The average annual precipitation is 360.3 mm, and its wettest month is April (with 47.9 mm). More than 90 percent of the water in this area is used for agricultural purpose. Farming is the basic occupation of the rural regions, with major crops, including cereals, fodder crops, potatoes and sugar beets. The socio-demographic information of study population (N = 8042) is shown in Table 5. In this study, the data include two datasets: 1. the data of death rate due to various types of malignancies (based on ICD Codes), diabetes mellitus, Alzheimer's and congenital anomalies, 2. The data of arsenic concentration. All data and information were obtained from Vice-chancellor for Health of Hamadan University of Medical Sciences over a period of five years (2016–2020).

**Table 5:** The socio-demographic factors of the study population.

Cities	Variables					
	Gender (n (%))		Habitation (n (%))			Age (mean (± std))
	Male	Female	Urban	Rural	Unknown	
Asadabad (n = 52)	27 (52)	25 (48)	37 (71)	15 (29)	0 (0)	57.36 (± 19.58)
Bahar (n = 432)	237 (55)	195 (45)	195(45)	227 (53)	10 (0.02)	63.47 (± 22.15)
Kabudarahang (n = 708)	384 (54)	324 (46)	115(16)	570 (81)	23 (0.03)	61.78 (± 23.11)
Famenin (n = 170)	95 (56)	75 (44)	54 (32)	78 (46)	38 (22)	64.42 (± 20.37)
Razan (n = 651)	382 (59)	269 (41)	95 (15)	546 (84)	10 (0.02)	67.51 (± 20.81)
Tuyserkan (n = 509)	278 (55)	231 (45)	219 (43)	285 (56)	5 (0.01)	59.19 (± 25.21)
Nahavand (n = 735)	454 (62)	281 (38)	328 (45)	378 (51)	31 (0.04)	63.82 (± 20.30)
Hamadan (n = 3575)	1939 (54)	1636 (46)	3020 (84)	523 (15)	32 (0.01)	67.05 (± 18.78)
Malayer (n = 1210)	708 (59)	502 (41)	686 (57)	462 (38)	62 (0.05)	61.56 (± 22.39)

The analysis of arsenic concentration in water samples was carried out by the experts of the Vice-chancellor for Health as follows: The water samples were collected in clean polyethylene bottles, and labelled for easy identification. The samples were filtered through a 0.45 µm membrane filter immediately after collection. Then, they were transferred to the reference laboratory at the shortest possible time, under cold chain conditions. The samples were refrigerated and stored at 4 °C until the measurement was taken. The pH of the samples was stabilized in the range of 0.2 ± 0.2 by nitric acid^39^. The analysis of the samples was performed using a Metrohm 797 VA Computrace Voltammograph (Metrohm, Switzerland) equipped with a voltammetric cell^40^.

All data were collected and cleanup in an Excel form. The data was determined using Stata software, and appropriate analytical statistical methods. Poisson regression was used to investigate the relationship between arsenic level and the death rate (per 100,000 population) due to various types of disease in Kabudarahang compared to the other cities of the Hamadan province. Using goodness of fit tests, the best model was selected for estimating the arsenic effect. All the tests were performed at the 95% confidence level.

## Data Availability

The datasets generated during and/or analyzed during the current study are available from the corresponding author on reasonable request.

## References

[1] Chikkanna, A., Mehan, L., Sarath, P., & Ghosh, D. Arsenic exposures, poisoning, and threat to human health: arsenic affecting human health. In *Environmental Exposures and Human Health Challenges *86–105 (IGI Global, 2019).

[2] de Souza ACM, de Almeida MG, Pestana IA, de Souza CMM (2019). Arsenic exposure and effects in humans: A mini-review in Brazil. Arch. Environ. Contam. Toxicol..

[3] Chen T, Yuan X, Su Y (2021). Impacts of petroleum exploitation activities on the speciation of inorganic arsenic in groundwater. Environ. Forensics.

[4] Shaji E, Santosh M, Sarath K, Prakash P, Deepchand V, Divya B (2021). Arsenic contamination of groundwater: A global synopsis with focus on the Indian Peninsula. Geosci. Front..

[5] Rahman MA, Hannan MA, Uddin MJ, Rahman MS, Rashid MM, Kim B (2021). Exposure to environmental arsenic and emerging risk of Alzheimer’s disease: Perspective mechanisms, management strategy, and future directions. Toxics.

[6] Ozturk M, Metin M, Altay V, Bhat RA, Ejaz M, Gul A, Unal BT, Hasanuzzaman M, Nibir L, Nahar K, Bukhari A (2021). Arsenic and human health: Genotoxicity, epigenomic effects, and cancer signaling. Biol. Trace Elem. Res..

[7] Amadi CN, Igweze ZN, Orisakwe OE (2017). Heavy metals in miscarriages and stillbirths in developing nations. Middle East Fertil. Soc. J..

[8] Xue Q, Ran Y, Tan Y, Peacock CL, Du H (2019). Arsenite and arsenate binding to ferrihydrite organo-mineral coprecipitate: Implications for arsenic mobility and fate in natural environments. Chemosphere.

[9] Shah AH, Shahid M, Khalid S, Natasha A, Shabbir Z, Bakhat HF, Murtaza B, Farooq A, Akram M, Shah GM, Nasim W (2020). Assessment of arsenic exposure by drinking well water and associated carcinogenic risk in peri-urban areas of Vehari, Pakistan. Environ. Geochem. Health.

[10] Barzegar R, Asghari Moghaddam A, Kazemian N (2015). Assessment of heavy metals concentrations with emphasis on arsenic in the Tabriz plain aquifers, Iran. Environ. Earth Sci..

[11] Keshavarzi B, Seradj A, Akbari Z, Moore F, Shahraki AR, Pourjafar M (2015). Chronic arsenic toxicity in sheep of Kurdistan province, Western Iran. Arch. Environ. Contam. Toxicol..

[12] Mosaferi M, Shakerkhatibi M, Dastgiri S, Jafar-abadi MA, Khataee A, Sheykholeslami S (2017). Natural arsenic pollution and hydrochemistry of drinking water of an urban part of Iran. Avicenna J. Environ. Health Eng..

[13] Sadeghi F, Nasseri S, Mosaferi M, Nabizadeh R, Yunesian M, Mesdaghinia A (2017). Statistical analysis of arsenic contamination in drinking water in a city of Iran and its modeling using GIS. Environ. Monit. Assess..

[14] Dhillon AK (2020). Arsenic contamination of India’s groundwater: A review and critical analysis. Arsen. Water Resour. Contam..

[15] Yang M-H, Zang Y-S, Huang H, Chen K, Li B, Sun G-Y, Zhao X-W (2014). Arsenic trioxide exerts anti-lung cancer activity by inhibiting angiogenesis. Curr. Cancer Drug Targets.

[16] Pokhrel D, Bhandari B, Viraraghavan T (2009). Arsenic contamination of groundwater in the Terai region of Nepal: An overview of health concerns and treatment options. Environ. Int..

[17] Guo H, Wen D, Liu Z, Jia Y, Guo Q (2014). A review of high arsenic groundwater in Mainland and Taiwan, China: Distribution, characteristics and geochemical processes. Appl. Geochem..

[18] Stopelli E, Duyen VT, Mai TT, Trang PT, Viet PH, Lightfoot A, Kipfer R, Schneider M, Eiche E, Kontny A, Neumann T (2020). Spatial and temporal evolution of groundwater arsenic contamination in the Red River delta, Vietnam: Interplay of mobilisation and retardation processes. Sci. Total Environ..

[19] Ravenscroft P, Brammer H, Richards K (2011). Arsenic Pollution: A Global Synthesis.

[20] Wang W, Cheng S, Zhang D (2014). Association of inorganic arsenic exposure with liver cancer mortality: A meta-analysis. Environ. Res..

[21] Khanjani N, Jafarnejad A-B, Tavakkoli L (2017). Arsenic and breast cancer: A systematic review of epidemiologic studies. Rev. Environ. Health.

[22] Chen C-J, Chuang Y-C, Lin T-M, Wu H-Y (1985). Malignant neoplasms among residents of a blackfoot disease-endemic area in Taiwan: High-arsenic artesian well water and cancers. Cancer Res..

[23] Basu A, Ghosh P, Das JK, Banerjee A, Ray K, Giri AK (2004). Micronuclei as biomarkers of carcinogen exposure in populations exposed to arsenic through drinking water in West Bengal, India: A comparative study in three cell types. Cancer Epidemiol. Prev. Biomark..

[24] Lin M-H, Li C-Y, Cheng Y-Y, Guo H-R (2022). Arsenic in drinking water and incidences of leukemia and lymphoma: Implication for its dural effects in carcinogenicity. Front. Public Health.

[25] Liaw J, Marshall G, Yuan Y, Ferreccio C, Steinmaus C, Smith AH (2008). Increased childhood liver cancer mortality and arsenic in drinking water in northern Chile. Cancer Epidemiol. Prev. Biomark..

[26] Tsai S-M, Wang T-N, Ko Y-C (1999). Mortality for certain diseases in areas with high levels of arsenic in drinking water. Arch. Environ. Health Int. J..

[27] Li X-L, Zhan R-Q, Zheng W, Jiang H, Zhang D-F, Shen X-L (2020). Positive association between soil arsenic concentration and mortality from Alzheimer’s disease in mainland China. J. Trace Elem. Med Biol..

[28] Marie C, Léger S, Guttmann A, Marchiset N, Rivière O, Perthus I, Lémery D, Vendittelli F, Sauvant-Rochat MP (2018). In utero exposure to arsenic in tap water and congenital anomalies: A French semi-ecological study. Int. J. Hyg. Environ. Health.

[29] Ahmed SM, Noble BN, Joya SA, Ibn Hasan MOS, Lin PI, Rahman ML, Mostofa G, Quamruzzaman Q, Rahman M, Christiani DC, Kile ML (2019). A prospective cohort study examining the associations of maternal arsenic exposure with fetal loss and neonatal mortality. Am. J. Epidemiol..

[30] Navas-Acien A, Spratlen MJ, Abuawad A, LoIacono NJ, Bozack AK, Gamble MV (2019). Early-life arsenic exposure, nutritional status, and adult diabetes risk. Curr. Diab.Rep..

[31] Mahram M, Shahsavari D, Oveisi S, Jalilolghadr S (2013). Comparison of hypertension and diabetes mellitus prevalence in areas with and without water arsenic contamination. J. Res. Med. Sci..

[32] Baris D, Waddell R, Beane Freeman LE, Schwenn M, Colt JS, Ayotte JD, Ward MH, Nuckols J, Schned A, Jackson B, Clerkin C (2016). Elevated bladder cancer in Northern New England: the role of drinking water and arsenic. J. Natl. Cancer Inst..

[33] Saint-Jacques N, Brown P, Nauta L, Boxall J, Parker L, Dummer TJ (2018). Estimating the risk of bladder and kidney cancer from exposure to low-levels of arsenic in drinking water, Nova Scotia, Canada. Environ. Int..

[34] de Lanerolle-Dias M, Lanerolle P, Atukorala S, de Silva A (2015). Urbanisation, dietary patterns and body composition changes in adolescent girls: A descriptive cross sectional study. BMC Nutr..

[35] Momenyan S, Sadeghifar M, Sarvi F, Khodadost M, Mosavi-Jarrahi A, Ghaffari ME, Sekhavati E (2016). Relationship between urbanization and cancer incidence in Iran using quantile regression. Asian Pac. J. Cancer Prev..

[36] Navipour E, Neamatshahi M, Barabadi Z, Neamatshahi M, Keykhosravi A (2019). Epidemiology and risk factors of Alzheimer’s disease in Iran: A systematic review. Iran. J. Public Health.

[37] Zahnd WE, James AS, Jenkins WD, Izadi SR, Fogleman AJ, Steward DE, Colditz GA, Brard L (2018). Rural–urban differences in cancer incidence and trends in the United States. Cancer Epidemiol. Biomark. Prev..

[38] Halimi L, Bagheri N, Hoseini B, Hashtarkhani S, Goshayeshi L, Kiani B (2020). Spatial analysis of colorectal cancer incidence in Hamadan Province, Iran: A retrospective cross-sectional study. Appl. Spat. Anal. Policy.

[39] Vidosavljevic M, Puntaric D, Gvozdic V, Vidosavljevic D, Juric D, Begovic L (2022). Assessment of arsenic in hair of the inhabitants of East Croatia—Relationship to arsenic concentrations in drinking water. Water.

[40] Abadi M, Zamani A, Parizanganeh A, Khosravi Y, Badiee H (2018). Heavy metals and arsenic content in water along the southern Caspian coasts in Iran. Environ. Sci. Pollut. Res..

